# Mechanisms of Cadmium-Induced Testicular Injury: A Risk to Male Fertility

**DOI:** 10.3390/cells11223601

**Published:** 2022-11-14

**Authors:** Waseem Ali, Yonggang Ma, Jiaqiao Zhu, Hui Zou, Zongping Liu

**Affiliations:** 1 College of Veterinary Medicine, Yangzhou University, Yangzhou 225009, China; 2 Jiangsu Co-Innovation Center for Prevention and Control of Important Animal Infectious Diseases and Zoonoses, Yangzhou 225009, China; 3 Joint International Research Laboratory of Agriculture and Agri-Product Safety of the Ministry of Education of China, Yangzhou University, Yangzhou 225009, China

**Keywords:** cadmium, Sertoli cells, Leydig cells, testicular toxicity, infertility

## Abstract

Cadmium is a heavy toxic metal with unknown biological functions in the human body. Over time, cadmium accretion in the different visceral organs (liver, lungs, kidney, and testis) is said to impair the function of these organs, which is associated with a relatively long biological half-life and a very low rate of excretion. Recently studies have revealed that the testes are highly sensitive to cadmium. In this review, we discussed the adverse effect of cadmium on the development and biological functions of the testis. The Sertoli cells (SCs), seminiferous tubules, and Blood Testis Barrier are severely structurally damaged by cadmium, which results in sperm loss. The development and function of Leydig cells are hindered by cadmium, which also induces Leydig cell tumors. The testis’s vascular system is severely disturbed by cadmium. Cadmium also perturbs the function of somatic cells and germ cells through epigenetic regulation, giving rise to infertile or sub-fertile males. In addition, we also summarized the other findings related to cadmium-induced oxidative toxicity, apoptotic toxicity, and autophagic toxicity, along with their possible mechanisms in the testicular tissue of different animal species. Consequently, cadmium represents a high-risk factor for male fertility.

## 1. Introduction

Sperm are distinct cells produced in one animal and released, then enter another animal to transfer their genetic material to produce offspring by sexual reproduction [1]. However, before transition, the sperm undergo molecular changes in their sugars, lipids, and proteins. Glycoproteins and polysaccharides form an interaction between the sperm and its external milieu known as the glycocalyx [2]. The High levels of soluble glycohydrolases and glycosyltransferases found in the luminal fluid are played an essential role in the modification of sperm surface glycoconjugates [3]. A negative change in the external sperm surface charge during epididymal maturation has been shown [4]. It is thought that the increase in negative charge is due to variations in sugar moieties, particularly the incorporation of negatively-charged sialic acid [5]. The acquisition of progressive sperm motility, a basic requirement for the ability to undergo hyperactivation when exposed to capacitating conditions, is one of the functional changes occurring during epididymal maturation. In addition, related to the capacitation process, sperm also undergo an increase in protein tyrosine phosphorylation as part of their transit through the epididymis to bind to the zona pellucida and undergo the acrosomal reaction to become able to fuse with the oolemma to fertilize the oocyte [6]. Interestingly, numerous studies have demonstrated the considerable contribution of the sperm, which contributes both its DNA and its whole structure to embryo development [7]. During fertilization, sperm-specific proteins and factors trigger Ca^2+^ oscillations to stimulate the oocyte. In contrast, the sperm centriole directs both oocyte and sperm nuclei to produce the zygote nucleus, and sperm DNA structures free RNAs and chromatin can be altered to activate or deactivate gene expression necessary in embryo development [8]. These interactions show that all the physiological and biochemical changes that take place during epididymal transit allow the sperm to undergo capacitation.

Infertility is a serious global health problem that disquiets millions of couples worldwide [9]. Male factors contribute to infertility in about 40–50% [10]. Reduced sperm count, aberrant sperm morphology, and poor sperm motility are common underlying reasons for poor male fertility that are seen in semen analysis [11]. However, 15% of males with normal spermograms suffer infertility. Therefore, additional sperm parameters may be essential in diagnosing male infertility [12]. In this disorder, the rate of embryonic development, implantation, pregnancy, and in vitro and in vivo fertilization is all negatively correlated with DNA fragmentation, which affects the sperm, causing infertility [13]. Male age (over 35), lifestyle (drinking and smoking), some types of cancer, pathological and genetic variables, environmental toxins, and others may all contribute to DNA fragmentation in sperm [14]. DNA fragmentation occurs in sperm following oxidative stress-induced apoptosis [15]. Apoptosis is a critical regulator of germ cell growth during spermatogenesis [16]. However, ejaculated sperm can also show apoptotic signs [17]. According to research, more apoptotic sperm was reported in the semen of infertile patients than in fertile donors [18]. The most significant characteristics of apoptosis are activation of caspase, chromatin condensation, DNA fragmentation, changes in phosphatidylserine location within the plasma membrane, and disturbance of mitochondrial membrane potential (MMP) [19,20,21,22]. 

Environmental toxins can cause various diseases, including infertility [23]. Cadmium is one of the most toxic metals, has no known useful biological function, and is a major public health risk, especially reproductive toxicity [24,25]. Many investigations have revealed that mammalian testes are very sensitive against cadmium, which causes toxicity in male reproductive organs, particularly the testicles and sperm parameters, because of their active cell division and metabolism [26,27,28,29,30]. Before the fusion with the oocyte, sperm must undergo a number of challenges during activation to achieve fertilization competence [31] because these physiological processes for fertilization are generally activated by the activation of ion channels on the sperm membrane [32]. Sperm has a specific cation channel (CatSper) which acts as the main source of intracellular Ca^2+^ and can cause several Ca^2+^ dependent responses (progesterone-induced acrosome reaction, chemotaxis, and sperm motility/viability), whereas the sperm-specific potassium channel accounts for membrane potential hyperpolarization. CatSper and (KSper) are both crucial channels for regulating the physiologic function of sperm and, consequently, male fertility [33]. Cadmium is hypothesized to interfere with protein tyrosine phosphorylation by competing for binding calmodulin with calcium [34]. Cadmium has also been shown to reduce axonal protein phosphorylation by increasing membrane lipid peroxidation. Cadmium has been found to have a negative effect on sperm metabolism, which is assumed to be mediated through the inhibition of glycogen phosphorylase, magnesium-dependent ATPase, glucose-6-phosphatase, and succinate dehydrogenase [35]. Whereas the impairment in the functional activity of these channels (expression, permeability, and decreased blood pH), as well as reduced viability and motility of sperm, is caused by cadmium concentration that causes male infertility [33].

**Figure 1:**
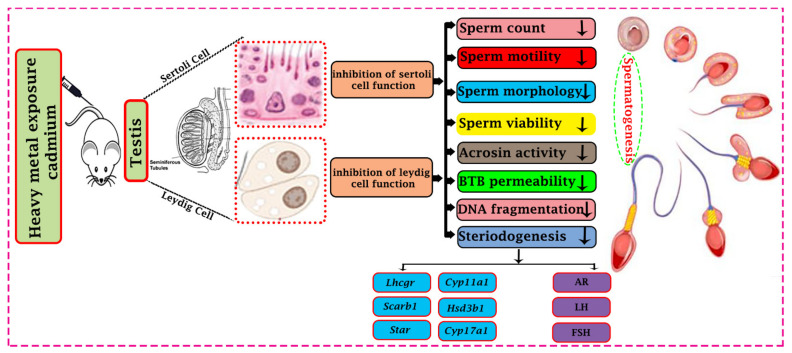
Effect of cadmium toxicity on the functional activity of a male reproductive organ. Cadmium causes abnormal development and disturbs testicular cells’ functional activity (Sertoli cells and Leydig cells). Effects of Cadmium prohibit the different physiological processes like steroidogenesis and spermatogenesis. Cadmium reduces steroidogenic function by down-regulating the steroidogenic gene’s expression (*Lhcgr*, *Cyp11a1*, *Scarb1*, *Hsd3b1*, *Star* and *Cyp17a1*) and proteins like androgen, luteinizing hormone, and follicle-stimulating hormone (AR, LH, and FSH). Cadmium affects different developmental parameters of sperm and may lead to a reduction of spermatogenesis. In addition, cadmium causes impairment in the immune response through the Blood Testis Barrier (BBB) permeability. All these changes may impair the ability to reproduce.

According to evidence from epidemiological research, cadmium and male infertility/sterility are positively associated. The other studies revealed that the 60 infertile adult males (40 with oligospermia and 20 with azoospermia) had higher serum and semen cadmium levels than the 40 normal sperm control [36]. A high level of cadmium in blood was found in 501 cases of infertile couples in Rockville, USA, representing that cadmium has reproductive toxicity at environmentally relevant levels. Varicocele in males commonly shows increased cadmium buildup in the testicular blood system and an overall rise in sperm cell apoptosis in the testis [37]. High-qualified research included in meta-analysis can produce more reliable outcomes. High level of cadmium in semen causes male infertility, according to a meta-analysis of 11 studies that included 1093 infertile individuals and 614 control [38]. The study of 50 healthy males discovered that blood cadmium concentrations positively correlated with decreased sperm motility and teratozoospermia [39]. In 1020 males, the levels of three heavy metals—including cadmium, arsenic, and lead—as well as markers of oxidative stress were measured in their urine. The results demonstrated that the advanced levels of arsenic, cadmium, and lead were inversely associated with positive semen and increased with markers of oxidative stress [40]. In males, cadmium can have endocrine-disrupting consequences. In a study of 2,286 males (18 years and older), the blood cadmium levels were found to be negatively impacted by total testosterone (TT) and sex hormone-binding globulin (SHBG) [41]. Kresovic et al.’s data from the 1999 National Health and Nutrition Examination Survey (NHANES) were used to measure the relationship between blood cadmium and SHBG in men, and there was found a positive correlation between them [42]. In the present review, we pay attention to the effects of cadmium on testis and sperm parameters which ultimately lead to male infertility (Figure 1), and also discuss the various mechanisms involved in cadmium-induced oxidative stress, apoptosis, and autophagy, and steroidogenic variations in testicular tissue. 

## 2. Effect of Cadmium on Spermatogenesis

### 2.1. Effect of Cadmium on SCs

Mammalian testis comprises two different compartments, (the seminiferous epithelial tissue, SCs hold together to support sperm production), and (the mesenchymal compartment), where the interstitial cells called Leydig cells (LCs) secrete androgens and insulin-like peptide 3 (INSL3) to regulate the male reproductive developmental tract, testicular lineage, and sperm production. During the fetal and neonatal periods, SCs are essential for developing the testicular cord [43,44]. The tubular structure is disrupted, and adult Leydig cells’ subsequent growth (ALCs) growth in the adult testis is substantially impeded when SCs in neonatal mice testes are deleted [45]. SCs in adulthood tests are essential for maintaining sperm production, and their absence causes the testis to stop producing germ cells [45]. Additionally, SCs secrete anti-Müllerian hormone (AMH) in the fetus, which triggers Mullerian regression [46]. The number of SCs rises exponentially in mice and humans during fetal development, slows after birth, and reaches adult levels at puberty [47,48,49,50]. 

Bakheet et al. have demonstrated that the development of SCs is impacted by cadmium during the prenatal and neonatal periods (Table 1). Gene expression (*Dhh and Fshr*) was down-regulated by a single intraperitoneal injection of low-dose cadmium into mice on GD12, although their numbers were unaltered [51]. In pregnant and nursing rats, subcutaneous exposure to cadmium (1–2 mg/kg) causes SCs vacuolation and deletion of germ cells in the adult seminiferous epithelium [52]. Cadmium inhibits immature SCs proliferation, causes mitochondria, DNA damage, and cell death in the testicles, and causes abnormal and apoptotic ultrastructures in SCs [53]. In vitro SC-germ cell co-culture system, cadmium inhibits the contact between newborn SCs and germ cells via p38MAPK signaling, leading to increased germ cell apoptosis [54]. A daily dose of 1 mg/kg of cadmium administered orally to rats for 28 days caused severe ultrastructural changes in adult SC [55]. The cytoplasm of SCs in rats given a single dosage of cadmium (3 mol/kg) showed vacuolation. According to biological data, cadmium at 0.5–20 M following changed expression alters the structure of F-actin in human SCs. The SC actin cytoskeleton is damaged in vitro by the actin-regulating proteins Arp3 and Eps8 [56].

### 2.2. Effect of Cadmium on Sperm Development 

Cadmium affects the developmental parameters of sperm (Table 2). Single dosages of cadmium (0.67–1.1 mg/kg) for 7 days disrupted the seminiferous epithelium in sperm [60]. Rat sperm count, motility, and viability declined after 28 days of oral cadmium (5 mg/kg) treatment [68]. The number of germ cells was reduced, and the seminiferous tubules of the testes were disordered in mice exposed to cadmium (0.2 mg/kg, sc) for 15 days [69]. Cadmium (1.15 mg/kg, IP) administration for 56 days resulted in substantially damaged seminiferous tubules in adult male rats [70]. Mice treated to cadmium (3 mg/kg, sc, once per week) for four weeks similarly have their seminiferous tubules shrink, their germ cells decrease, and their multinucleated giant cells grow [71].

### 2.3. Effects of Cadmium on Sperm Maturation

Cadmium affects how mature sperm function (Table 1). After in vitro treatment, cadmium significantly decreased sperm motility and maturation in human and animal sperm. Short-term (30 min) cadmium concentration had no effect on sperm motility, but it markedly decreased the egg in vitro fertilization rate and postponed the early stages of embryonic development in mice, showing that cadmium has epigenetically-based effects. Human sperm’s motility and forward movement are likewise decreased by cadmium [30]. The studies have shown that low doses of cadmium concentration 50 g/day, nearly 30 to 60 fold less than short-term doses, negatively affect reproduction. These effects include interruptions in the histology of the epididymis and testis, injury to spermatogenesis, a decrease in sperm motility, a change in sperm morphology, and a reduction in the rate of acrosome reaction [30,76].

### 2.4. Effect of Cadmium on Blood Testis Barrier Formed by SCs

Mammalian testes have specialized junctions between neighboring (SCs) that are closed to the basement membrane of seminiferous tubules that build up the Blood Testis Barrier, which is very important for male germ cell development and protection [77]. The Blood Testis Barrier is targeted by different environmental pollutants [78,79,80]. It causes down-regulation of SCs tight and gap junction forming proteins such as claudin 5, claudin 11, occludin, Cx43, and ZO-1, which are the main reasons for the damaging of Blood Testis Barrier permeability, which perturbs spermatogenesis and finally indicates to male infertility [81]. 

The cadmium has been postulated to impact Blood Testis Barrier negatively. In a rat model, cadmium triggers Blood Testis Barrier disruption. In mice and humans, cadmium damages Blood Testis Barrier by causing SCs actin filament disintegration [56,82]. According to mechanistic research, cadmium perturbs the Blood Testis Barrier in rat testes in vivo via increasing TGF-b3, which stimulates the p38 MAPK signaling pathway [83,84], which in turn triggers the p38 MAPK signaling pathway. It is interesting to note that cadmium simultaneously activates the JNK pathway to increase 2-macroglobulin to counteract its negative impacts as a particular JNK inhibitor. According to Wang et al., the JNK signaling pathway is a protective mechanism in the SCs following cadmium treatment, which may increase cadmium-induced damage to the Blood Testis Barrier [82]. The expression of p-FAK Tyr397 and p-FAK Tyr576 are down-regulated by cadmium during the cadmium-mediated Blood Testis Barrier interruption, and cadmium-induced actin cytoskeleton disordering at the SCs Blood Testis Barrier is caused by dislocation of Arp-3 and Eps-8 [80]. In addition, by decreasing the expression of occludin and urokinase plasminogen activator proteins for 8 h, cadmium treatment of SCs can stop SC’s tight junction construction without significantly increasing cytotoxicity [60]. A non-receptor protein tyrosine kinase called focal adhesion kinase controls Blood Testis Barrier [85]. Focal adhesion kinase controls the tight junction proteins (such as occludin and ZO-1) [86]. Cadmium can regulate focal adhesion kinase expression [87]. These findings provide insight into the therapeutic approach to modulating cadmium-induced male infertility, such as through the p38 MAPK inhibitor or in combination with the JNK activator and p-FAK Tyr397 and p-FAK Tyr576, respectively.

### 2.5. Effect of Cadmium on Leydig Cells 

The integrity of Leydig cells is important for spermatogenesis. Leydig cells are responsible for the secretion and production of androgen hormones. However, cadmium decreases the circulating testosterone, causes disorganization of mitochondria of Leydig cells, cell viability, increased lipid peroxidation, DNA damage, and damage to the testicular blood vessels [63]. Laskey et al. observed that decreased testosterone levels appeared before morphological changes in the testis after cadmium injection [64]. In male fetuses and their testicular progeny, androgens facilitate the development of the internal and external genitalia; androgens promote the development of the vas deferens, seminal vesicles, and Wolff’s duct in male mammals [88]. Fetal and adult Leydig cell function and growth are impacted by cadmium (Table 1). Male fetal Leydig cell steroidogenic genes and proteins expression *(Lhcgr*, *Scarb1*, *Star*, *Cyp11a1*, *Hsd3b1*, and *Cyp17a1),* fetal testicular testosterone synthesis, and fetal Leydig cell number were reduced in rats after a single dose of cadmium (0.25, 0.5, and 1.0 mg/kg, IP) [51]. After the cadmium induction in the rat testis during the perinatal period, the development of adult Leydig cells is suppressed, with an increase in the number of immature Leydig cells (cells expressing Srd5a1) and a decline in cAMP/PKA signaling pathway and down-regulation of steroidogenic testosterone enzymes [89]. Adult male rats exposed to cadmium displayed elevated PGF2a and lowered serum testosterone and StAR-controlled levels. The expressions of *Lhcgr*, *Scarb1*, *Star*, *Cyp11a1*, *Hsd3b1*, *Hsd17a1*, and *Hsd17b3* were all significantly suppressed in adult male mice exposed to cadmium (0.5 or 1.0 mg/kg, single IP dose). Adult rats given a high dose of cadmium (0.45 mg/kg, sc) had reduced blood testosterone levels, decreased sex organ weights, and HSD3B1 and HSD17B3 gene activities.

Furthermore, in vitro research demonstrates that cadmium also inhibits testosterone production and Leydig cell DNA integrity. In R2C tumor Leydig cells, cadmium concentration dependently reduces cAMP and suppresses dihydrolipoamide dehydrogenase expression. Primary Leydig cells exposed to 10, 20, and 40 μM cadmium for 24 h also showed increased DNA lower testosterone selection and replication. Additionally, cadmium damages vascular cells and causes Leydig cell tumors [90]. Future studies are needed to determine whether cadmium is efficient in affecting Leydig cell development during fetal and pubertal stages.

### 2.6. Cadmium-Induced Apoptotic Changes in Testis

Apoptosis is a concept of programmed cell death and involves various morphological and biochemical actions, including cell shrinkage, DNA fragmentation, membrane blabbing, chromatin decondensation, and the formation of apoptotic bodies [91,92]. In the testes, the death of germ cells by apoptosis in a highly controlled method is essential for the normal process of spermatogenesis to maintain the fertility potential of males [93]. However, various published studies have shown that spermatogenic potency decreases or increases when this controlled cell death mechanism is inhibited by cadmium, which impairs male fertility. 

Cadmium promotes apoptosis in different cells and organs, and many signaling pathways (Mitogen-activated protein kinase pathway, Ca^2+^ pathway, nuclear factor-Kb pathway, and phosphatidylinositol-3-kinase) are involved in cadmium-induced apoptosis [94,95]. Apoptosis has been observed in the liver [96], lungs [97], fibroblast [98], and kidney [99]. In addition, in testis, this programmed cell death process has been investigated. McCloskey (2006) has demonstrated a specific process of cadmium’s effects on apoptosis; cadmium especially induces the death of early spermatogenic cells through apoptosis and does not affect other parameters, like the permeability of the Blood Testicular Barrier [100]. In rats, acute cadmium administration caused testicular apoptosis, as evidenced by the presence of TUNEL-positive cells that simultaneously declined the testicular weight [101]. Cadmium-induced testis showed increased activity of caspase 3 and 9, which increases the number of morphological signs of apoptosis, including chromatin decondensation and marginalization in primary spermatocytes, loss of the nuclear envelope in the spermatogonium, disruption of mitochondrial membrane potential (MMP), degradation of cytoplasmic organelles, and DNA distribution [102]. The testicles of rats [103], fish [104], and lizard [105] all showed evidence of cadmium-induced spermatogonia and spermatocyte apoptosis. The concentration-dependent effect of cadmium on human embryonic germ cell apoptosis was revealed by immune signaling detection of caspase 3 in testicular tissue [106]. In a concept of caspase-dependent apoptosis, caspase-3 acts as the executioner. However, apoptosis can also take place in a number of cells without the need for caspase [107]. The lack of caspase-3 immunoreactivity in apoptotic sperm recommends that the morphological and biochemical features of cadmium chloride-induced sperm apoptosis are not interceded by caspase activation. Rat testicular germ cells were found to be induced to undergo apoptosis after acute cadmium induction through endoplasmic reticulum pathways [108]. Some studies have demonstrated the role of mitochondrial pathways in inducing apoptosis in testicular germ cells through the dysregulation of the Bcl-2 protein, cytochrome c, tumor necrosis factor (TNF-α), and B cell lymphoma 2 (*Bcl-2*) genes, with simultaneously increased activities of caspase 3 and 7 in cadmium-induced in mice [93,109,110]. Additionally, a recent study demonstrates that in duck testicles, Bak-1 mRNA levels and caspase 3 are up-regulated, while Bcl-2 levels are down-regulated [111]. The caspase 8, caspase-3-p53 pathway may interact with the cadmium-induced intrinsic and extrinsic pathways of apoptosis during turtle spermatogenesis [112]. These studies demonstrate the complexity of the various apoptotic pathways through which cadmium induces testicular apoptosis in various species and clarify a common mechanism of cadmium induction apoptosis in testes.

### 2.7. Role of Autophagy in Cadmium-Induced Testicular Injury 

Autophagy is an evolutionary conserved catabolic process that degrades the cytoplasmic components in lysosomes and plays an important role in many physiological and pathological processes [113,114,115,116]. This degrading pathway supports cells in getting rid of unwanted and damaging substances, which plays a significant role in the complex process of spermatogenesis [117]. Autophagy is necessary for complete sperm fate, including spermatogenesis, sperm maturation, and fertilization. Autophagy contributes to nucleus remodeling, mitochondrial rearrangement, flagellum, and acrosome development during spermatogenesis [118,119]. These regulated physiological processes require cellular equilibrium between cytoplasmic breakdown and recycling. Human sperm autophagy-related proteins like a light chain (LC3), autophagy-related gene (ATG5, ATG7), PTEN-induced kinase (PINK1), beclin-1 (BECN1), sequestosome 1 (p62), mechanistic target of rapamycin (mTOR), and adenosine monophosphate activated protein kinase (AMPKa) and their upstream regulations are functionally active, implying that autophagy may govern sperm motility [120]. Furthermore, Ca^2+^ signals play an important role in endoplasmic reticulum stress-induced autophagy. Ca^2+^ is released from the endoplasmic reticulum to induce autophagy [121]. Many signaling pathways are known to regulate autophagy under hypoxic and low-energy conditions. However, the effect of cadmium-induced autophagy on the testis remains unclear. 

Autophagy can be used as a stress adaptation and protection mechanism. However, in some cases, over-activated autophagy will induce cell death under severe oxidative stress and metal toxicity [122]. The cadmium-induced autophagy causes impairment in many body cells, and autophagic cell death may induce physiologically significant injury [123]. Cadmium-induced has been extensively investigated in the kidney [124] and liver [125]. However, the testis is another essential organ impacted by cadmium because of its critical function in male reproduction, and the toxic effects of cadmium concentration are a great clinical concern. The study showed that different degrees of cadmium concentration could cause testicular damage. Testicular cadmium decreases in spermatogenic cells and mature sperm, indicating a decrease in spermatogenic potential, which surely impacts reproductive health [126]. Autophagy can minimize the secretion of reactive oxygen and apoptosis, reducing the severity of cadmium-induced damage. However, the protective effect of autophagy on the body are somewhat limited because cadmium can cause irreversible cell damage by causing autophagic death when overall by interfering with intercellular communication through gap junctions, and autophagy can amplify this effect, resulting in increased damage [127]. Other studies have revealed that cadmium can boost inflammatory reactions in the testicles. When rats were exposed to cadmium, they could not produce functional metallothionein, which normally reduces inflammatory responses. The number of cells undergoing autophagy increases as a result of high levels of inflammatory cytokines produced by cadmium absorption [128]. In addition, some have argued that autophagic cells were highly susceptible to cadmium, resulting in enhanced autophagy-induced organ injury in response to cadmium concentration [129]. Cadmium promotes lysosomal acidification in vivo and in vitro by inducing the lysosomal-associated membrane protein 2 (LAMP2) and the lysosomal hydrolase cathepsin B and increases lysosomal degradation potential. However, cadmium suppresses Rab7 protein expression, resulting in defective fusion of the autophagosomes with lysosomes [130,131]. Cadmium concentration elevated the expression of the autophagy proteins LC3B, Beclin1, sequestosome 1 (SQSTM1/p62), and its autophagy active form LC3B-II in the testis. These data suggested cadmium concentration induced oxidative stress and impaired autophagic degradation, which caused testicular damage. Weng et al. proposed that cadmium-induced autophagy could be mediated in vitro by a calcium signaling pathway; thus, calcium-sensing-receptor activation might be an important trigger for autophagic cell death that resulted in testicular damage and proposed that calcium-sensing-receptor protein expression is significantly higher in cadmium exposed testes [132]. Collectively, these findings suggest that activating normal autophagy may be beneficial; however, cadmium-induced defective autophagy increases the risk of testicular injury. However, it is important to determine the further in detail molecular mechanism of autophagy in cadmium-induced injury in the testis.

## 3. Conclusions

**Figure 2:**
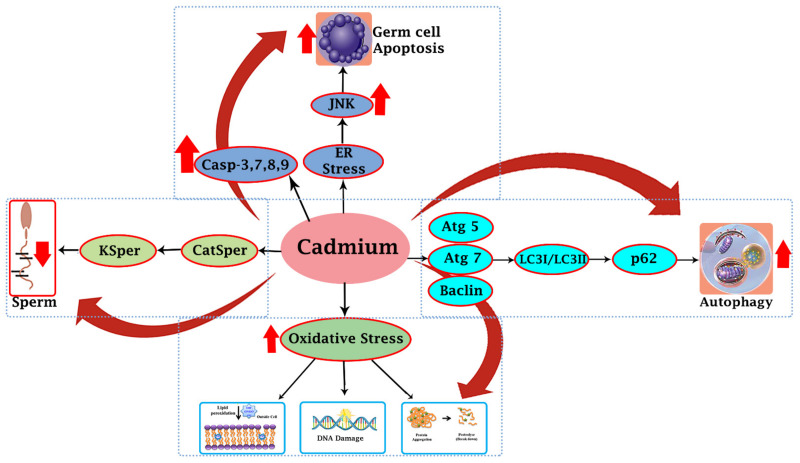
Potential mechanisms for cadmium-induced testicular toxicity. Up-regulation of caspase (3, 7, 8, & 9) by cadmium outcomes is testicular germ cells’ apoptosis; cadmium may also up-regulate the phosphorylation of (JNK) factors, which provoke apoptosis of germ cells through the endoplasmic reticulum stress pathway. As well as, cadmium may up-regulate the expression of autophagy-related proteins leading to high activation autophagy, which causes cell death and testicular injury. Cadmium may induce oxidative stress and cause to increase in lipid peroxidation, DNA damage, and protein damage. Cadmium may down-regulate the Ca^2+^ and K^+^ channels, and reduction in (CatSper and KSper) functioning by cadmium may lead to poor quality of sperm.

According to this review, cadmium is a strong testicular toxicant that changes the various physiological processes in the testicular tissue of different animal species exposed to cadmium through environmental and occupational sources. Cadmium has been shown to decrease testicular biochemical function and steroidogenic activities in the testis, induce oxidative stress, germ cell apoptosis, and cadmium-induced autophagy (Figure 2). These findings are openly related to decreasing male reproductive potential. 

Studies have exposed the connection of the sperm-specific cation channel (CatSper) and (KSper) in cadmium-induced male infertility, the Nrf2 signaling pathway in cadmium-induced oxidative stress, and MMP/p38 MAPK pathways in cadmium-induced apoptosis, and as well as enhanced autophagy in cadmium-induced testicular injury. However, further detailed mechanism is still needed more attention. Therefore, more inclusive research using animal models which are closely phylogenetic with human and reflecting real situations of cadmium exposure, dose and routes of administration are required to define the exact mechanism associated with cadmium mediated alterations of different physiological processes in the testis. So, this study contributes a scientific base for future research to develop safe and effective approaches against cadmium toxicity in the testis. 

## Figures and Tables

**Table 1:** Effect of cadmium on SC and Leydig cell of the testis.

Cell	Species	Concentration	Action	Reference
**SC**	Human	0.5–20 µM	Increase BTB disturbance	[56]
**SC**	Mouse	Inhaled 0.006 M	Increase Mitochondrial changing	[57]
**SC**	Rat	0–40 µM, 0.3 mg/100 g	Increase Apoptosis	[54,58]
**SC**	Mouse	0.5 μL/g	Increase DNA damage	[59]
**SC**	Rat	0.1–10 µM	Increase Cytoplasmic damage	[60]
**SC**	Rat	1.0 mg/kg	Reduction of cell migration, damage of cytoskeletal proteins	[51]
**SC**	Rat	1.5 mg/kg	Reduced cell viability	[61]
**SC**	Rat	1.5 mg/kg	Reduced sperm count	[62]
**Leydig cell**	Wistar Rat	10–40 µM & 2.5 mg/kg	Reduction of Testosterone hormone secretion	[63,64,65]
**Leydig cell**	Rat	6–8 g/day in food	Reduction of Steroidogenic gene expression	[66]
**Leydig cell**	Rat	1.5 mg/kg	Reduction of Leydig cell number	[61]
**Leydig cell**	Mouse	0.015 g/L orally	Leydig cell tumor	[67]

**Table 2:** Effect of cadmium toxicity on sperm parameters.

Species	Concentration	Sperm Parameters	Description	Reference
**Human**	5–100 mg/L	Morphology	Reduced	[72]
**Rat**	0–8 mg/Kg	Motility	Reduced	[73]
**Human**	3.0 and 1.0 µg/L	Viability	Reduced	[74]
**Rat**	5–100 mg/L	Concentration	Reduced	[75]
**Mouse**	0–250 µM	Acrosome reaction	Reduced	[33]

## Data Availability

Not applicable.
